# Innate and Adaptive Immune Responses to Clinical Hyaluronic Acid Fillers

**DOI:** 10.1111/jocd.70292

**Published:** 2025-07-10

**Authors:** Joshua S. T. Hooks, Brenda Yang, David R. Maestas, James I. Andorko, Anna Ruta, Joscelyn C. Mejias, Sean H. Kelly, Christopher K. Hee, Jennifer H. Elisseeff

**Affiliations:** ^1^ Department of Biomedical Engineering, Translational Tissue Engineering Center, Wilmer Eye Institute Johns Hopkins University Baltimore Maryland USA; ^2^ Bloomberg~Kimmel Institute for Cancer Immunotherapy, Sidney Kimmel Comprehensive Cancer Center Johns Hopkins University School of Medicine Baltimore Maryland USA; ^3^ Allergan Aesthetics An AbbVie Company Irvine California USA

**Keywords:** biomaterials, flow cytometry, hyaluronic acid, immune response

## Abstract

**Background:**

Crosslinked hyaluronic acid (HA)‐based hydrogels are commonly used as dermal fillers where they interact with surrounding tissues including host stromal and immune cells. HA fillers are widely used for aesthetic applications, with products designed with varying properties depending on their indication. Although HA fillers have been demonstrated to have a strong biocompatibility profile, a small subset of patients’ experiences delayed‐onset adverse events hypothesized to be inflammatory and allergy‐related outcomes such as delayed‐onset hypersensitivity.

**Aims:**

The overall goal of this study was to evaluate the innate and adaptive immune response to two clinically available HA filler formulations.

**Methods:**

Using multiparametric flow cytometry, we characterized the immune response to Juvèderm Volbella (VYC‐15 L) and Juvèderm Ultra 3 (SGD‐30XP) in a murine quadricep muscle resection that enables implantation of larger volumes and exposure to muscle and adipose.

**Results:**

Presence of the implanted HA filler increased recruitment of immune cells, specifically antigen presenting macrophages, eosinophils, and gamma‐delta (γδ) T cells to the injury site compared to no implant (saline) controls. Comparing the two materials, VYC‐15 L increased interleukin 17a (IL17a) production by lymphocyte subsets at the injury site and induced higher levels of circulating IgE relative to SGD‐30XP and saline controls.

**Conclusion:**

Overall, these results provide insights into the immune response to HA fillers and how different formulations may alter the immune outcomes.

## 1 Introduction

Hyaluronic acid (HA), also commonly referred to as hyaluronan, is a glycosaminoglycan found in the extracellular matrix (ECM) throughout all tissues of the body and is particularly abundant in the skin and joints [1]. Crosslinked HA‐based hydrogels have become one of the most common injectable dermal fillers for cosmetic use [2]. The biophysical properties of these HA hydrogels give them a space‐filling effect ideal for providing volume and smoothing wrinkles [3]. HA has generally been described as highly biocompatible, making it ideal for biomedical application, but HA regulates a host of cellular functions [4]. Depending on the concentration, molecular weight, and state of degradation, HA can play multiple, and sometimes contradictory, regulatory roles on processes such as immune activation and angiogenesis [5, 6]. As it degrades, HA may act as a ligand for multiple cellular receptors, including CD44 and several toll‐like receptors, facilitating immune cell recruitment and activation [6, 7].

In clinical use, HA fillers and hydrogels are well tolerated, with over 2 million individuals receiving cosmetic HA dermal fillers annually [8, 9]. Many currently available dermal fillers contain HA and 1,4‐Butanediol diglycidyl ether (BDDE) as a crosslinking agent. Even though various fillers contain the aforementioned ingredients, such fillers can vary significantly in their composition and properties. Differences in composition can include variation of the molecular weight of HA used (low [<500 kDa] vs. lower [0.5 to 1 MDa] vs. higher [> 1 MDa] molecular weight), the amount of crosslinking, and the conditions in which the crosslinking is performed. Juvèderm Ultra 3 (SGD‐30XP) and Juvèderm Volbella (VYC‐15 L) are examples of commercially available HA fillers containing BDDE as the crosslinking agent that have different rheological properties and composition. SGD‐30XP is primarily comprised of higher molecular weight HA (> 1 MDa). Alternatively, VYC‐15 L is comprised of a mix of higher (> 1 MDa) and lower molecular weight HA (0.5 to 1 MDa), relative to SGD‐30XP, which allows for more flexibility to achieve the desired physical properties [10, 11]. The differences in composition and physical properties (such as elastic moduli (G'), cohesivity (resistance to compression and stretching), and water uptake) among HA fillers can lead to different abilities to perform their function as space‐filling biomaterials, such as ease of injection in superficial versus deeper sites [12], altering the volumizing/filling effect, or reducing short‐term water uptake [11, 13]. These properties can be modulated to tailor the outcomes for different indications [14].

Although HA fillers are generally well tolerated, in very rare cases some patients have developed delayed adverse reactions at the implant site, which may occur months to years after the injection procedure. These adverse events have been described as edema/swelling, nodules or lumps, granulomatous foreign body reaction, and/or general inflammation at the site of previous filler injections [15, 16, 17, 18]. These late‐onset reactions are often associated with a “secondary insult” or pre‐existing condition, such as infection or illness; however, the etiology of these events is not well defined [19, 20, 21, 22]. Although studies have failed to reveal a clear HA‐driven mechanism for these reactions [23], it has been hypothesized that these events may be a result of an allergic response or hypersensitivity to the fillers. More specifically, delayed‐onset responses to HA fillers have been hypothesized to possibly be either type I hypersensitivity or type IV delayed hypersensitivity. Type 1 hypersensitivity is an allergy‐related, antigen‐specific immune response mediated by IgE antibodies [24]. Type IV delayed hypersensitivity is T cell mediated rather than antibody mediated, and is further categorized based on the types of innate immune cell populations recruited to the site [25].

Though it has been hypothesized that these adverse reactions to HA fillers may be hypersensitivity responses, the local cellular response to the fillers has not been described in detail. Here, we use multiparametric flow cytometry to broadly characterize the immune response to HA filler implantation following a volumetric muscle loss (VML) model. The VML model creates a defect by partial surgical resection of the quadriceps femoris, allowing the biomaterial to be implanted into the defect space where it lies adjacent to muscle and adipose. The tissue damage from surgical resection increases the immune response to the material, and the VML model enables the excision of a control tissue (quadricep muscle) in the no implant (saline) condition. We implanted two HA fillers, VYC‐15 L and SGD‐30XP, into the tissue injury and characterized the innate and adaptive immune profile over 6 weeks.

## 2 Materials and Methods

### 2.1 Biomaterial Implantation

All animal procedures were performed with approval by Johns Hopkins University Institutional Animal Care and Use Committee (ACUC). VYC‐15 L and SGD‐30XP were provided by Allergan for implantation. Mice received bilateral removal of muscle from their quadriceps as described in previous publications [26, 27]. Briefly, 7–10‐week‐old female C57BL/6 WT mice (Jackson Labs) were anesthetized under 3.0%–4.0% isoflurane in oxygen at a 200 cc/min flow rate and maintained at 2.0% isoflurane. Lower limbs were shaved and cleaned of excess hair using 70% ethanol and kimwipes. A 1 cm incision in the skin and fascia above the quadricep muscle group was made using surgical scissors. A 3 x 3 x 4 mm section of the muscle was removed along the mid‐belly of the muscle group. Defects were filled immediately with 50 μL of either VYC‐15 L or SGD‐30XP gels. For control surgeries, 50 μL of sterile 1x Dulbecco's phosphate buffered saline (Gibco) was dispensed into the wound. Wounds were closed with 3–4 wound clips, and the procedure was repeated on the contralateral limb. Following surgery, mice were immediately given subcutaneous injections of Rimadyl (5 mg/kg) for pain management.

### 2.2 Flow Cytometry

#### 2.2.1 Tissue Digestion

At the respective time points (1, 3, or 6 weeks) quadricep muscle and residual filler implants were harvested, diced into 1 mm pieces, then enzymatically digested using 0.5 mg/mL Liberase TL (Sigma) plus 0.2 mg/mL DNase I (Roche) in RPMI media on a shaker at 50–75 rpm, 37°C, for 45 min. Resulting digested tissue was filtered through a 70 μm cell strainer followed by a 40 μm cell strainer to create a single‐cell suspension for surface staining. This single‐cell suspension was stained as is for the pan‐immune panel on the Aurora Cytometer (Cytek Biosciences) and the myeloid panel on the Attune NxT Flow Cytometer (Thermo Fisher Scientific).

For intracellular staining, density gradient centrifugation via Percoll (GE Healthcare) was used to enrich lymphocyte cells and remove debris from the muscle samples. In summary, an 80%, 40%, and 20% Percoll solution were made by diluting Percoll with culture media (RPMI‐1640). Enzymatically digested quads/filler were resuspended in 80% Percoll in a 15 mL conical tube. 40% Percoll was carefully pipetted on top of the 80% layer, followed by a 20% Percoll layer. The conical tubes were centrifuged at 2100 g for 45 min with acceleration and deceleration set to 0, causing cells and debris to separate across the Percoll layers based on density. Lymphocytes, residing between the 80% and 40% Percoll layers, were carefully pipetted and transferred to clean tube for wash steps. Following washing, cells were stimulated with Cell Stimulation Cocktail plus protein transport inhibitors (eBioscience) and then diluted in culture media supplemented with 5% fetal bovine serum for 4 h. Cells were washed, stained for surface markers, fixed/permeabilized with Cytofix/Cytoperm (BD), and then stained for intracellular cytokines and transcription factors with the intracellular staining panel on the Attune NxT Flow Cytometer (Thermo Fisher Scientific).

All antibody panels are listed in Tables 1, 2, 3.

**TABLE 1:** Antibodies for Aurora Pan‐Immune Panel.

Fluorophore	Marker	Clone	Catalog	Manufacturer
BV421	Siglec‐F	E502440	562 681	BD
SuperBright436	CD19	1D3	62‐0193‐82	ThermoFisher
Pacific Blue	Ly6g	1A8	127 612	BioLegend
BV605	CD45	30‐F11	103 140	BioLegend
BV650	Ly6C	HK1.4	128 049	BioLegend
BV711	γδ TCR	GL3	563 994	BD
BV750	B220	RA3‐6B2	103 261	BioLegend
BV785	F4/80	BM8	123 141	BioLegend
BB515	cKit	2B8	564 481	BD
SparkBlue550	CD3	17A2	100 260	BioLegend
PerCP	MHC‐II	M5/114.15.2	107 624	BioLegend
BB700	CD8a	53–6.7	566 409	BD
PerCP‐eFluor710	CD86	GL1	46‐0862‐80	ThermoFisher
PE	CD301b	URA‐1	146 804	BioLegend
PE‐Dazzle594	CD11c	N418	117 348	BioLegend
PE‐Cy7	CD200R3	Ba13	142 212	BioLegend
APC	CD206	C068C2	141 708	BioLegend
AF647	NK1.1	PK136	108 720	BioLegend
AF700	CD11b	M1/70	101 222	BioLegend
Zombie NIR	Viability		423 106	BioLegend
APC‐Fire750	CD9	MZ3	124 814	BioLegend
APC‐Fire810	CD4	GK1.5	100 480	BioLegend

**TABLE 2:** Antibodies for Attune Myeloid Panel.

Fluorophore	Marker	Clone	Catalog	Manufacturer
BV421	CD86	GL1	105 031	BioLegend
BV510	Ly6C	HK1.4	128 033	BioLegend
BV605	CD45	30‐F11	103 139	BioLegend
BV711	CD11b	M1/70	101 241	BioLegend
FITC	CD9	MZ3	124 808	BioLegend
PerCP‐Cy5.5	Ly6G	1A8	127 616	BioLegend
PE	CD301b	URA‐1	146 804	BioLegend
PE/Dazzle 594	MHCII	M5/114.15.2	107 648	BioLegend
PE‐Cy7	F4/80	EMR1	123 114	BioLegend
APC	CD206	C068C2	141 708	BioLegend
AF700	CD11c	NF18	117 320	BioLegend
eFluor780	Viability		65‐0865‐14	ThermoFisher

**TABLE 3:** Antibodies for Attune Intracellular Staining (ICS) Panel.

Fluorophore	Marker	Clone	Catalog	Manufacturer
BV421	FoxP3	MF‐14	126 419	BioLegend
BV510	CD45	30‐F11	563 891	BD
BV605	NK1.1	PK136	108 739	BioLegend
BV711	CD8a	53–6.7	100 747	BioLegend
AF488	CD3	GK1.5	100 423	BioLegend
PerCP‐Cy5.5	CD19	6D5	115 534	BioLegend
PE	IL‐4	11B11	504 104	BioLegend
PE/Dazzle 594	γδ TCR	GL3	331 226	BioLegend
PE‐Cy7	CD4	GK1.5	100 422	BioLegend
APC	IFNγ	XMG1.2	505 810	BioLegend
AF700	IL‐17a	TC11‐18H10.1	506 914	BioLegend
eFluor780	Viability		65‐0865‐14	ThermoFisher

All gating schemes are detailed in Figures S2–S5.

#### 2.2.2 Serum ELISA

Six weeks following VML surgery, blood was collected via the submandibular vein into serum separator tubes (BD 365967). Total IgE levels in serum were determined using an enzyme‐linked immunosorbent assay (ELISA) kit (BD 555248, BD 550534) following the manufacturer's protocol.

#### 2.2.3 Histology

During tissue harvest, quadriceps and any residual material implant were immediately fixed in 10% neutral buffered formalin for 36 h, followed by ethanol‐based dehydration and embedding in paraffin wax. Seven μm thick sections were rehydrated and stained with hematoxylin and eosin (H&E) according to the manufacturer's instructions. Sections were imaged with a slide scanner (Hamamatsu—C12000).

## 3 Results

### 3.1 Innate and Adaptive Immune Cells Infiltrate HA Fillers

**FIGURE 1:**
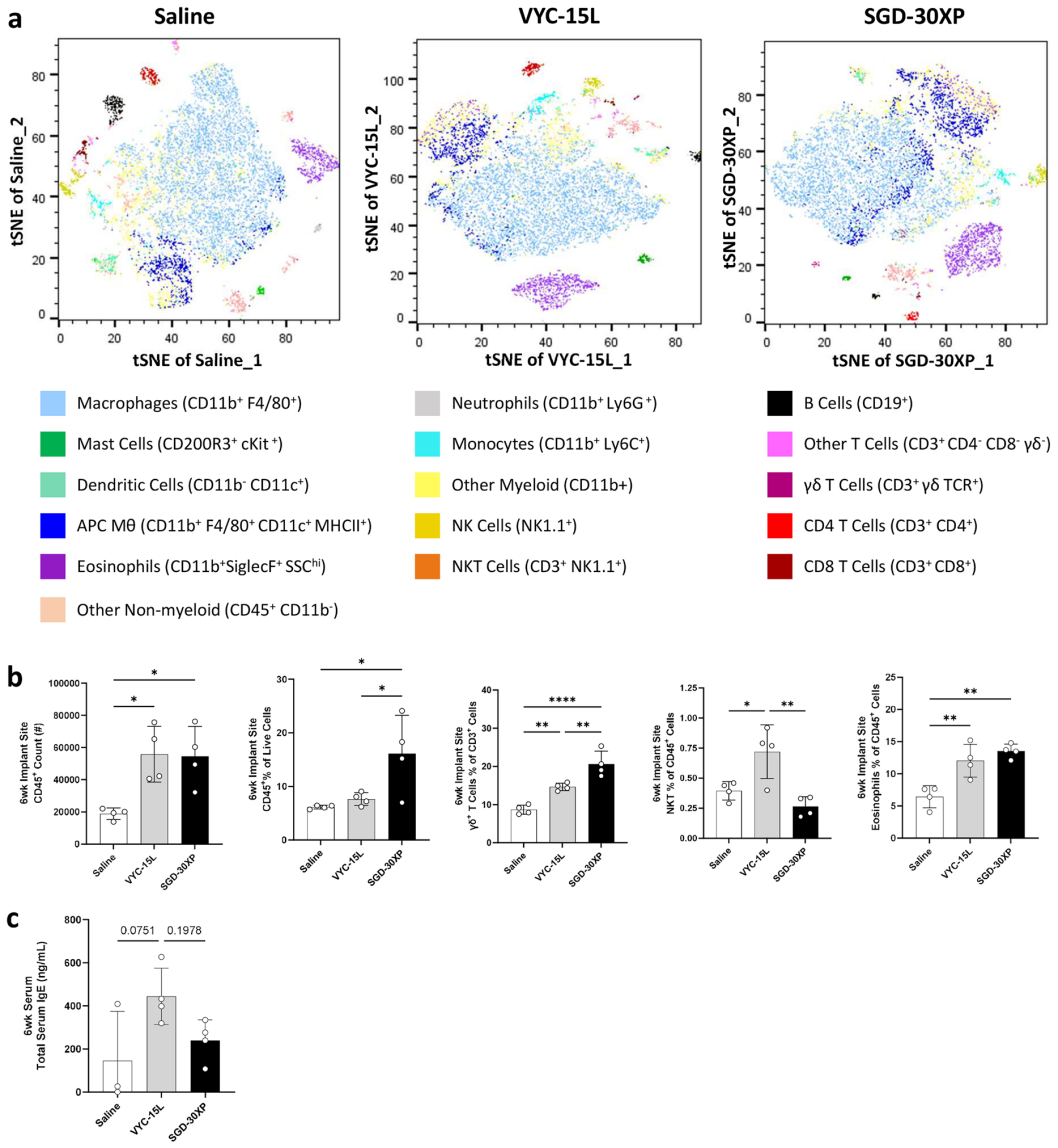
HA fillers polarize immune profile toward an allergy‐like response at 6 weeks following VML. Mice underwent volumetric muscle loss (VML) surgery and VYC‐15 L or SGD‐30XP HA fillers were implanted in the resection. Control mice were treated with a physiological saline. (a) Immune profiles as tSNE plots from spectral flow cytometry. (b) Bar plots of key immune cell populations shifted by HA filler implant compared to saline control. (c) Increased serum IgE detected in VYC‐15 L condition via ELISA. (Statistics) Data are mean ± SD, *n* = 4, ****p < 0.0001, ***p < 0.001, **p < 0.01, and *p < 0.05 by one‐way ANOVA with Tukey's multiple comparisons test.

To evaluate the immune response to crosslinked HA fillers, we performed a bilateral VML injury on wild‐type mice (C57BL/6) and implanted the fillers, VYC‐15 L or SGD‐30XP. Saline‐treated wounds were used as a control. We first evaluated the broad immune response using a pan‐immune multiparameter spectral flow cytometry panel 6 weeks after implantation. Results were visualized using a t‐distributed stochastic neighbor embedding (tSNE) plot. All live, CD45^+^ cells from all samples within a condition were concatenated and down sampled to 12 000 events. Then, tSNE was performed for 1000 iterations. Color‐coded cell populations were determined by expression of classically defined markers for each immune cell population (Figure 1a).

Implantation of the fillers increased the number of CD45^+^ immune cells recruited to the site after 6 weeks (Figure 1b). The filler VYC‐15 L did not significantly increase the proportion of immune cells among all live cells at the implant site, suggesting a corresponding increase of non‐immune cells (CD45^−^) in the VYC‐15 L condition (Figure 1b). Both HA fillers significantly increased the percentage of CD3^+^ γδ^+^ T cells in the implant site relative to saline (Figure 1b). VYC‐15 L alone led to increased NKT (NK1.1^+^, CD3^+^) cells at the implant site (Figure 1b). Both HA fillers upregulated eosinophils (SiglecF^+^, SSC^hi^) (Figure 1b), a myeloid population associated with allergy. Since eosinophils were present around the fillers in the muscle, we evaluated serum immunoglobulin E (IgE) levels. VYC‐15 L increased serum IgE levels as measured by ELISA compared to injury alone or SGD‐30XP (Figure 1c). Other notable shifts in immune cell populations with filler implantation include a downregulation in monocytes and neutrophils as a percent of live CD45^+^ cells (Figure S1).

### 3.2 HA Fillers Skew Immune Cell Phenotypes and T Cell Cytokine Production

**FIGURE 2:**
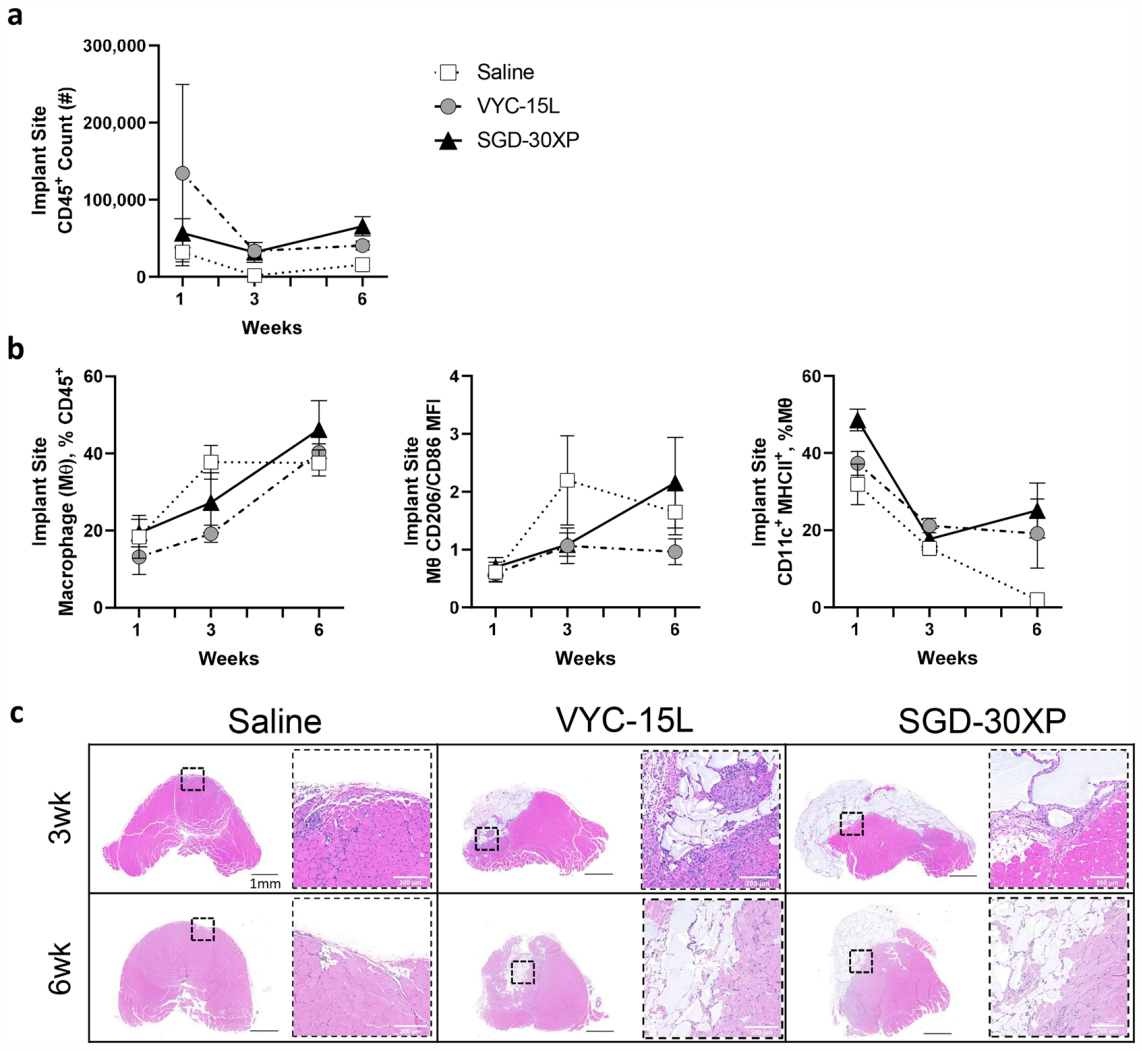
HA fillers enhance immune cell recruitment and increased macrophage antigen presentation markers. (a) HA fillers increase immune cell recruitment to the wound at all follow‐up time points. (b) Macrophages as a percent of immune cells increase over time across all conditions. No consistent shifts in M1 vs. M2 polarization (CD206/CD86 MFI) in either HA filler. Markers for antigen presentation (CD11c & MHCII) are upregulated on macrophages in HA filler conditions at the 6‐week time point. (c) H&E staining reveals residual filler at the wound site at both 3 and 6 weeks following implant. VYC‐15 L filler had less remaining material compared to SGD‐30XP at both follow‐up time points. Scale bar, 1 mm. (Statistics) Data are mean ± SD, *n* = 4.

We further characterized the phenotypic subsets and cytokine expression of immune populations at the 1‐, 3‐, and 6‐week time points after implantation. The presence of HA fillers increased the numbers of immune cells in the muscle at all time points (Figure 2a). Histologically, the HA fillers were visible 6 weeks after implantation; however, the volume of VYC‐15 L in histological cross‐sections appeared visibly less than SGD‐30XP (Figure 2c). This could be due to either increased degradation or increased spreading of the VYC‐15 L filler.

Macrophage phenotype, characterized by CD206:CD86 MFI ratio and antigen presentation markers such as CD11c and MHCII, shifted across the time of implantation (Figure 2b). At the 6‐week time point, the proportion of macrophages with antigen presentation markers increased in both HA filler conditions relative to saline (Figure 2b). The polarization of the macrophages also shifted in the presence of the SGD‐30XP filler at the 6‐week time point where there was an elevated CD206:CD86 MFI ratio relative to the saline and VYC‐15 L conditions (Figure 2b), indicative of an alternatively activated, M2‐like macrophage phenotype.

**FIGURE 3:**
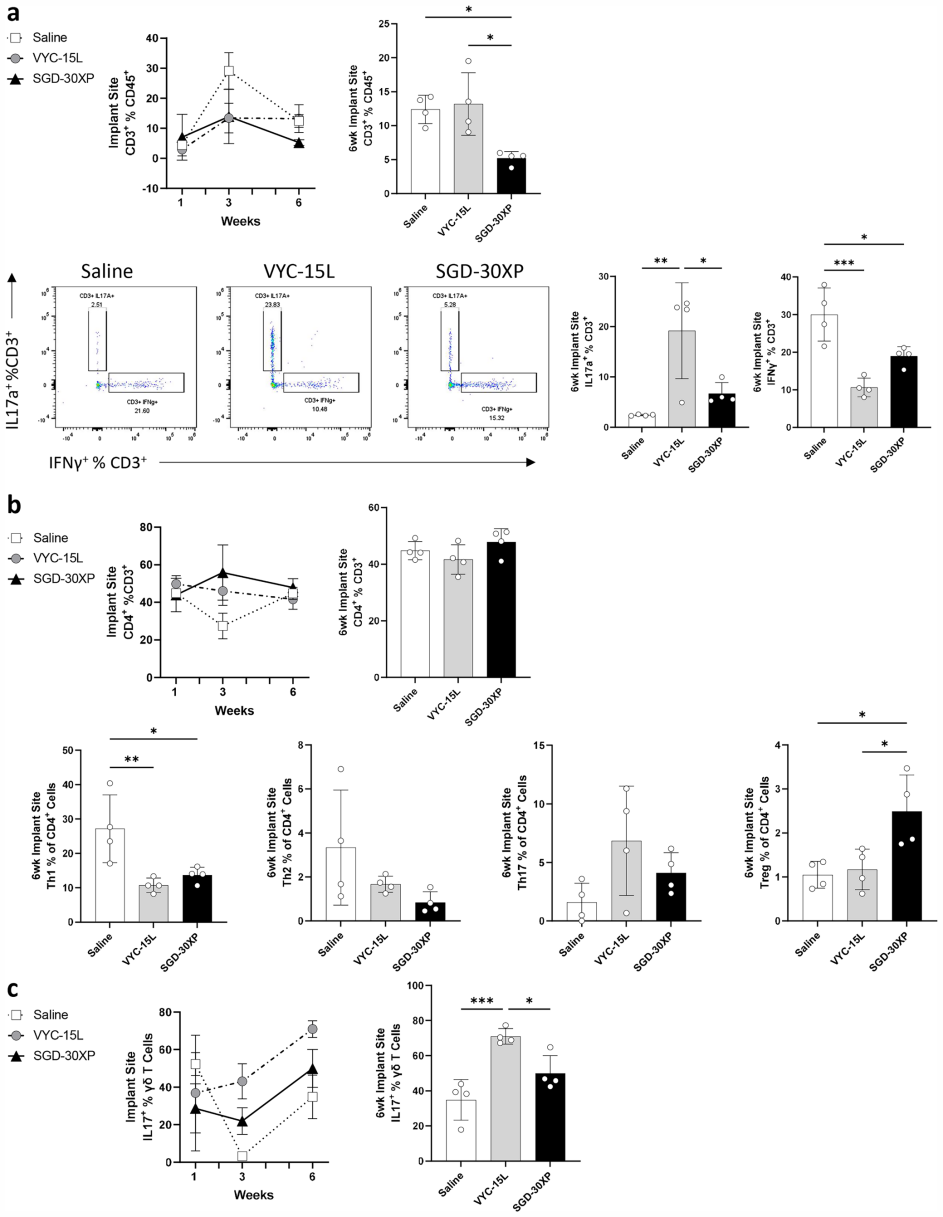
Differential cytokine production by local T cells following HA filler implant over course of wound healing. (a) At 6 weeks following VML, T cells were upregulated in the VYC‐15 L condition. T cells in the VYC‐15 L condition at 6 weeks following VML significantly increased IL17a expression. Both HA fillers downregulated T cell production of IFNγ. (b) There was not a difference in expression of overall CD4^+^ T helper cells throughout wound healing, but T helper cell subsets did shift significantly. IFNγ producing T helper cells (Th1) were significantly downregulated by HA fillers and FoxP3^+^ T regulatory cells (Tregs) were upregulated by SGD‐30XP at 6 weeks following VML. (c) VYC‐15 L upregulated IL17a^+^ γδ T cells, seen significantly at the 6‐week time point. (Statistics) Data are mean ± SD, *n* = 4, ***p < 0.001, **p < 0.01, and *p < 0.05 by one‐way ANOVA with Tukey's multiple comparisons test.

Additionally, at the 6‐week time point, there was a significant increase in the frequency of T cells in the VYC‐15 L filler compared to SGD‐30XP (Figure 3a). T cells in the VYC‐15 L filler significantly upregulated IL17a production relative to saline and SGD‐30XP, while the proportion of IFNγ‐producing T cells significantly decreased in the presence of both fillers (Figure 3a). To determine the cause of the cytokine expression differences, we explored various T cell subsets. Though the frequency of CD4^+^ T cells at 6 weeks did not change in both filler conditions relative to saline, there was a significant reduction in the IFNγ producing subset (Th1), a trend upward in IL17a‐producing subsets (Th17), and a trend downward in IL4‐producing T helper cells (Th2) (Figure 3b). The percentage of FoxP3^+^ T regulatory cells (Tregs) significantly increased in the SGD‐30XP condition compared to both saline and VYC‐15 L conditions (Figure 3b). In addition to CD4^+^ T cells, we demonstrated previously that γδ^+^ T cells can also contribute to IL17a production and the biomaterial response [28]. We found that the IL17a^+^ γδ^+^ T cells were upregulated by VYC‐15 L compared to SGD‐30XP and saline at both 3 and 6 weeks following VML (Figure 3c).

**FIGURE 4:**
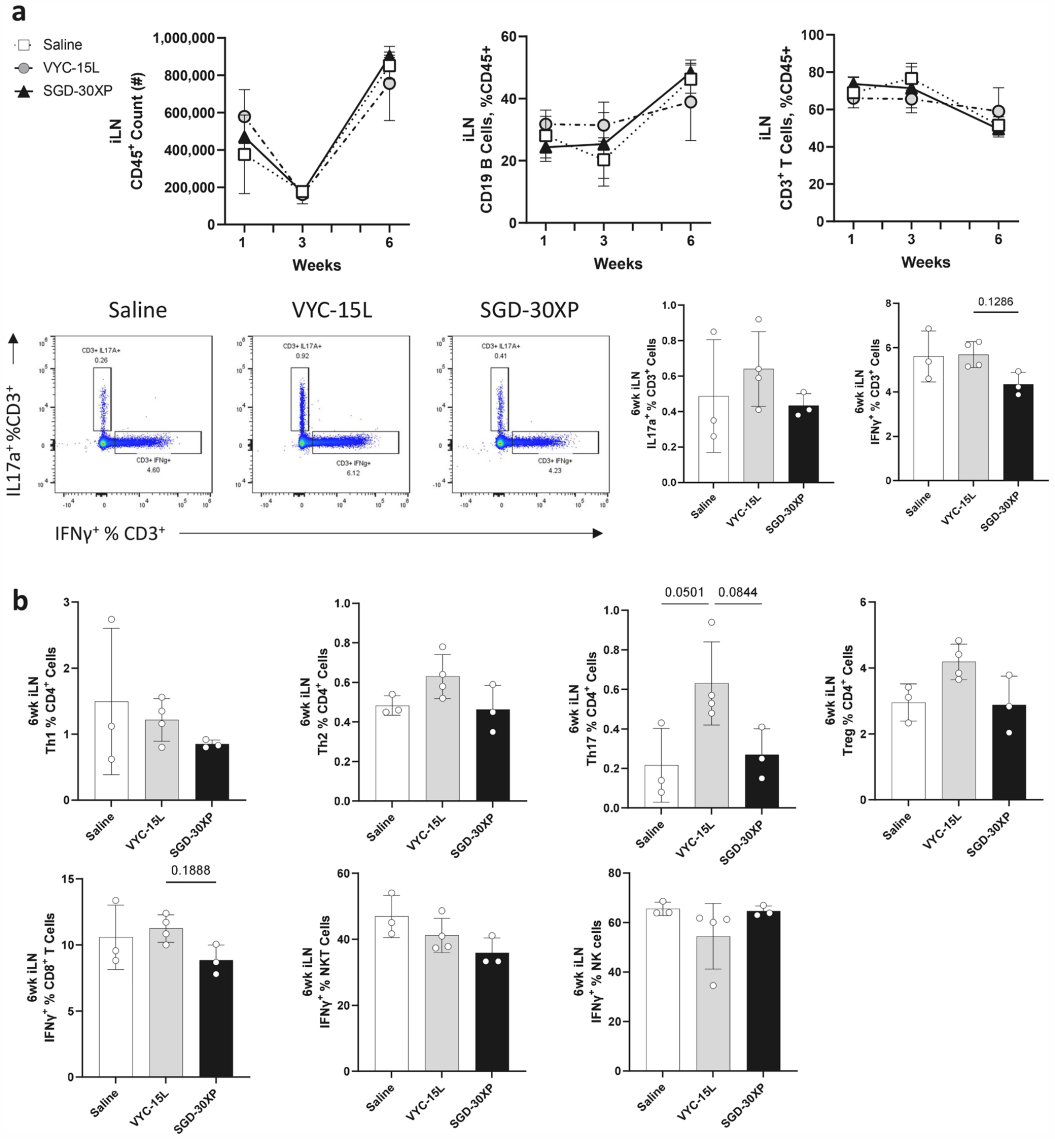
Immune expression of the draining inguinal lymph node (iLN) immune populations following VML and filler implantation. (a) Expression of immune cell populations was not significantly shifted by HA filler implant in the iLN. IL17a and IFNγ producing subsets were not significantly changed across all CD3^+^ cells. (b) Immune cell subsets reveal an increase in IL17a^+^ T helper cells (Th17) in the VYC‐15 L filler condition and a general downward trend in IFNγ producing lymphocytes (Th1, CD8^+^, and NKTs) in the SGD‐30XP filler condition compared to VYC‐15 L. (Statistics) Data are mean ± SD, *n* = 3–4, one‐way ANOVA with Tukey's multiple comparisons test.

In the draining inguinal lymph node (iLN), there were similar total immune cell counts and frequency of T and B cells across all conditions at the 1‐, 3‐, and 6‐week time points (Figure 4a). Looking at all T cells in the iLN (CD3^+^ cells), there was not a significant shift in IL17a production and a small trend downward in IFNγ production for the SGD‐30XP condition (Figure 4a). VYC‐15 L filler upregulated Th17 cells in the iLN, while SGD‐30XP was associated with a downward trend in IFNγ production across most IFNγ producing immune cell populations (Figure 4b).

## 4 Discussion

HA is broadly well tolerated in the body and has been shown to be pro‐regenerative in a variety of models. Both un‐crosslinked HA molecules and crosslinked HA hydrogels have been evaluated in a variety of applications for their impact on tissue regeneration. For example, HA has been shown to play an overall beneficial role in a variety of wound healing models including lung transplantation [29], myocardial infarction [30], osteoarthritis [31], and skin healing [32]. In those models, HA‐induced keratinocyte activation and angiogenesis, which contributed to tissue survival and wound closure. Additionally, HA regulates lymphatic vasculature as it degrades, which has been associated with improved resolution of inflammation and non‐fibrotic healing [33, 34, 35]. Generally, high molecular weight HA has been associated with immunosuppression while low molecular weight HA is associated with inflammatory signaling [7, 36], but tissue regeneration outcomes are model and formulation dependent [11, 37, 38]. Furthermore, it is well documented that immune response with hydrogels are heavily influenced by factors such as stiffness, porosity, and degradability [39, 40, 41].

To better understand how HA fillers impact immune cell recruitment, retention, and phenotype, we examined a broad spectrum of immune cell subsets over 6 weeks following HA filler implantation. We reveal the involvement of multiple innate and adaptive immune populations that differentiate the response between the two HA fillers (VYC‐15 L and SGD‐30XP), as well as the overall response to HA material relative to the saline control. We identify key immune cells associated with an allergy‐like response that are upregulated in the presence of HA filler implantation. Furthermore, we demonstrate that the T cell cytokine production profile at the implant site is shifted in the presence of both HA fillers. VYC‐15 L exacerbated IL17a signaling at late stages of wound healing compared to SGD‐30XP, while both HA fillers decreased IFNγ signaling. Further testing is required to determine whether these cellular responses are generally applicable to all HA fillers or are specific to certain classes of HA fillers. These experiments could identify which material properties of the HA fillers may be contributing to certain immune responses.

The general HA‐induced shifts in the immune profile at 6 weeks can be associated with different outcomes. For example, macrophages that are skewed toward an M2‐like phenotype (Figure 2) are present in tissue fibrosis and the regenerative phase of wound healing, along with allergy and delayed hypersensitivity response [42, 43]. CD11c expression in antigen presenting cells such as macrophages relates to antigen presentation to T cells, which is critical for allergy progression [44, 45]. Both macrophage phenotypic shifts can contribute to eosinophil recruitment, which is typically present in both allergic responses and muscle injury and healing. Therefore, the results may be indicative of an allergic response, the natural healing response in muscle after an injury, or a combination of both.

Relevant to allergic responses, we find an increase in serum IgE antibodies in the mice implanted with VYC‐15 L filler. In the allergy response, mast cells and basophils will “decorate” themselves with IgE via high‐affinity Fc receptor (FcεRI) stimulating granulation and release of inflammatory mediators upon binding [46]. We did not see an increase in overall mast cell expression associated with either filler (Figure S1), but future studies can be designed to look for activation markers of mast cells associated with IgE binding. Both fillers had enhanced γδ^+^ T cells in their immune profile (Figure 1b). Among many other roles, γδ^+^ T cells have been shown to be able to either enhance or suppress IgE following antigen challenge [47, 48]. More broadly, IL17 production by Th17 cells and γδ^+^ T cells, as seen in VYC‐15 L (Figure 3), has been associated with the pathogenesis of allergic rhinitis [49, 50] and γδ^+^ T cells have been shown to recognize antigens such as pollen [51].

Type IV delayed hypersensitivity, which includes contact sensitivity, is T cell mediated, involving CD4^+^, CD8^+^, γδ^+^, and NK1.1^+^ T cells [52]. There are four primary subsets of type IV hypersensitivities, with type IVb associated with Th2 lymphocytes and strong eosinophil recruitment [25]. Subsets of NKTs have been shown to initiate contact sensitivity and proliferate upon contact with antigen [53]. Similar to their role in other allergy models, γδ^+^ T cell subsets can have conflicting roles in either suppressing or exacerbating contact sensitivity [54, 55, 56]. While our results suggest an immune phenotype similar to type IV hypersensitivity (Figure 1b), additional testing such as intradermal allergy testing is needed to validate. Other recent studies have failed to induce an allergy response to HA using general allergic screenings, suggesting late‐onset inflammation following HA filler implant is not type IV hypersensitivity [23]. These studies have suggested that HA fillers may be acting as an adjuvant in late‐onset inflammatory cases when they are delivered containing endotoxins or if the wound becomes infected [57].

There was a notable downward shift in IFNγ production in both HA filler conditions (Figure 3a). IFNγ can contribute to the resolution of allergy‐related responses, and downregulation of Th1 cells has been associated with more severe allergic reaction [58, 59]. Tregs, uniquely upregulated in the SGD‐30XP condition (Figure 3b), can play a suppressive role in the activation and recruitment of dendritic cells, T helper cell subsets, and granulocytes, providing additional explanations for a potentially milder inflammatory response in SGD‐30XP treated mice compared to VYC‐15 L. It has also been shown that IFNγ expression increases after tissue injury and decreases as wound healing progresses [60, 61, 62]. The decrease in IFNγ with HA implantation may connect to a healing outcome. Further studies are needed to determine whether IFNγ plays a role in an allergic response and/or a healing response to the two HA fillers.

The HA fillers were not fully degraded by 6 weeks following implantation (Figure 2c). Degradation rate of HA fillers is influenced by a variety of factors, including the degree of crosslinking and the levels of endogenous hyaluronidase and reactive oxygen species [63]. While degradation was not directly measured in this study, the histology of the VYC‐15 L treated muscle indicated lower volume of remaining filler relative to the SGD‐30XP treated muscle (Figure 2c). VYC‐15 L is crosslinked with lower molecular weight HA compared to SGD‐30XP, which contributes to the differences in rheological properties [11]. Lower molecular weight HA chains are associated with increased inflammatory immune signaling and recruitment that can promote increased cell infiltration and possibly degradation [7]. Low molecular weight HA, produced during degradation, could explain the unique Th17‐skewed inflammatory response associated with the VYC‐15 L filler at the time points evaluated in this study. It is possible that on a time scale beyond 6 weeks, the SGD‐30XP filler could further degrade into lower molecular weight HA chains that induce a similar inflammatory phenotype. Longer term studies may elucidate how the hydrogel is incorporated into the tissue environment and how it impacts tissue vascularization, ECM production, and the potential for the development of a foreign body response (FBR) [64]. Evaluation of additional HA fillers with different compositions may help to identify additional contributing factors, such as specific material properties, or if this is a general effect of HA fillers. Lastly, these studies were performed in mice in a controlled laboratory environment that neglects the contributions of factors in the broader patient population, including age, history of infection, and diet, and that can all contribute to the immune environment.

## 5 Conclusion

This study demonstrates that the crosslinked HA fillers Juvèderm Volbella (VYC‐15 L) and Juvèderm Ultra (SGD‐30XP) induce unique innate and adaptive immune responses in a murine VML model. Both VYC‐15 L and SGD‐30XP fillers increased the recruitment of CD45^+^ immune cells to the implant site relative to the saline condition. Notably, VYC‐15 L led to increased IL17a production from γδ^+^ T cells and elevated systemic IgE levels, suggesting a potential link to delayed‐type hypersensitivity or allergy‐like responses. In contrast, SGD‐30XP induced a milder inflammatory phenotype, with increased M2‐like macrophages and Tregs at the implant site. These findings suggest that HA filler formulation influences immune dynamics at the implant site and provide insight into clinical observations of delayed inflammatory responses.

## Author Contributions

Joshua Hooks: conceptualization, investigation, writing – original draft, writing – review and editing, visualization, and supervision. Brenda Yang: formal analysis, investigation, writing – review and editing, and visualization. David Maestas: conceptualization, formal analysis, investigation, visualization, and supervision. James Andorko: conceptualization, investigation, and supervision. Anna Ruta: investigation and writing – review and editing. Joscelyn Mejias: formal Analysis and investigation. Sean Kelly: investigation and writing – review and editing. Christopher Kee: supervision and writing – review and editing. Jennifer Elisseeff: conceptualization, writing – review and editing, supervision, and funding acquisition.

## Conflicts of Interest

Allergan funded this study and provided the materials for this study. J.H.E. is an inventor on intellectual property related to biological scaffolds and inhibiting fibrosis. J.H.E. holds equity in Unity Biotechnology and Aegeria Soft Tissue. J.H.E. is a member of the scientific advisory boards of Tessera Therapeutics, HapInScience, and Font Bio. J.H.E. is a consultant for Vericel. C.K.H. is an employee of Allergan Aesthetics, an AbbVie company, and owns stock and stock options in AbbVie.

## Supporting information


**Figure S1.** Additional muscle immune cell populations at 6 weeks following VML. PMN, polymorphonuclear leukocyte. (Statistics) Data are mean ± SD, *n* = 4, ****p < 0.0001, ***p < 0.001, **p < 0.01, and *p < 0.05 by one‐way ANOVA with Tukey’s multiple comparisons test.
**Figure S2.** Gating scheme for Aurora Pan‐Immune panel on quadricep muscle.
**Figure S3.** Gating scheme for Attune myeloid panel on quadricep muscle.
**Figure S4.** Gating scheme for Attune ICS panel on quadricep muscle.
**Figure S5.** Gating scheme for Attune ICS panel on inguinal lymph node.

## Data Availability

All data is available in the manuscript and supplemental document.

## References

[1] J. R. E. Fraser , T. C. Laurent , and U. B. G. Laurent , “Hyaluronan: Its Nature, Distribution, Functions and Turnover,” Journal of Internal Medicine 242 (1997): 27–33. 9260563 10.1046/j.1365-2796.1997.00170.x

[2] G. Huerta‐ángeles , K. Nešporová , G. Ambrožová , L. Kubala , and V. Velebný , “An Effective Translation: The Development of Hyaluronan‐Based Medical Products From the Physicochemical, and Preclinical Aspects,” Frontiers in Bioengineering and Biotechnology 6 (2018): 1–13. 29868577 10.3389/fbioe.2018.00062 PMC5966713

[3] A. La Gatta , R. Salzillo , C. Catalano , et al., “Hyaluronan‐Based Hydrogels as Dermal Fillers: The Biophysical Properties That Translate Into a “Volumetric” Effect,” PLoS One 14 (2019): 1–17, e0218287. 10.1371/journal.pone.0218287 PMC6559669 31185059

[4] J. Alijotas‐Reig , M. Hindié , R. Kandhaya‐Pillai , and F. Miro‐Mur , “Bioengineered Hyaluronic Acid Elicited a Nonantigenic T Cell Activation: Implications From Cosmetic Medicine and Surgery to Nanomedicine,” Journal of Biomedical Materials Research. Part A 95 (2010): 180–190. 20564542 10.1002/jbm.a.32794

[5] M. Slevin , S. Kumar , and J. Gaffney , “Angiogenic Oligosaccharides of Hyaluronan Induce Multiple Signaling Pathways Affecting Vascular Endothelial Cell Mitogenic and Wound Healing Responses *,” Journal of Biological Chemistry 277 (2002): 41046–41059. 12194965 10.1074/jbc.M109443200

[6] J. Muto , Y. Morioka , K. Yamasaki , et al., “Hyaluronan Digestion Controls DC Migration From the Skin,” Journal of Clinical Investigation 124 (2014): 1309–1319. 24487587 10.1172/JCI67947 PMC3934161

[7] D. Jiang , J. Liang , and P. W. Noble , “Hyaluronan as an Immune Regulator in Human Diseases,” Physiological Reviews 91 (2011): 221–264. 21248167 10.1152/physrev.00052.2009 PMC3051404

[8] C. K. Hee , G. T. Shumate , V. Narurkar , A. Bernardin , and D. J. Messina , “Rheological Properties and In Vivo Performance Characteristics of Soft Tissue Fillers,” Dermatologic Surgery 41 (2015): S373–S381. 26618467 10.1097/DSS.0000000000000536

[9] I. B. Allemann and L. Baumann , “Hyaluronic Acid Gel (Juvéderm) Preparations in the Treatment of Facial Wrinkles and Folds,” Clinical Interventions in Aging 3 (2008): 629–634. 19281055 10.2147/cia.s3118 PMC2682392

[10] D. Eccleston and D. K. Murphy , “Juvéderm Volbella in the Perioral Area: A 12‐Month Prospective, Multicenter, Open‐Label Study,” Clinical, Cosmetic and Investigational Dermatology 5 (2012): 167–172. 23152693 10.2147/CCID.S35800 PMC3496328

[11] C. K. Hee and D. J. Messina , “In Vitro Inflammatory and Immune Response to Uncrosslinked Hyaluronic Acid (HA) and HA Fillers,” Journal of Immunology and Regenerative Medicine 17 (2022): 100065.

[12] A. T. Hillel , S. Unterman , Z. Nahas , et al., “Photoactivated Composite Biomaterial for Soft Tissue Restoration in Rodents and in Humans,” Science Translational Medicine 3 (2011): 93ra67. 10.1126/scitranslmed.3002331 PMC4652657 21795587

[13] H. Raspaldo , J. Chantrey , L. Belhaouari , et al., “Juvéderm Volbella With Lidocaine for Lip and Perioral Enhancement: A Prospective, Randomized, Controlled Trial,” Plastic and Reconstructive Surgery. Global Open 3 (2015): 1–8. 25878932 10.1097/GOX.0000000000000266 PMC4387143

[14] C. De La Guardia , A. Virno , M. Musumeci , A. Bernardin , and M. B. Silberberg , “Rheologic and Physicochemical Characteristics of Hyaluronic Acid Fillers: Overview and Relationship to Product Performance,” Facial Plastic Surgery 38 (2022): 116–123, 10.1055/s-0041-1741560. 35114708 PMC9188840

[15] S. Okada , R. Okuyama , H. Tagami , and S. Aiba , “Eosinophilic Granulomatous Reaction After Intradermal Injection of Hyaluronic Acid,” Acta Dermato‐Venereologica 88 (2008): 69–70. 18176759 10.2340/00015555-0324

[16] T. Bhojani‐Lynch , “Late‐Onset Inflammatory Response to Hyaluronic Acid Dermal Fillers,” Plastic and Reconstructive Surgery. Global Open 5 (2017): 1–7. 10.1097/GOX.0000000000001532 PMC5889432 29632758

[17] O. Bitterman‐Deutsch , L. Kogan , and F. Nasser , “Delayed Immune Mediated Adverse Effects to Hyaluronic Acid Fillers: Report of Five Cases and Review of the Literature,” Dermatology Reports 7 (2015): 12–14. 10.4081/dr.2015.5851 PMC4387334 25918619

[18] M. G. Turkmani , K. De Boulle , and W. G. Philipp‐Dormston , “Delayed Hypersensitivity Reaction to Hyaluronic Acid Dermal Filler Following Influenza‐Like Illness,” Clinical, Cosmetic and Investigational Dermatology 12 (2019): 277–283. 31118731 10.2147/CCID.S198081 PMC6501047

[19] A. Owczarczyk‐Saczonek and W. Placek , “The Immunogenicity of Hyaluronic Fillers and Its Consequences,” (2021): 921–934. 10.2147/CCID.S316352 PMC8291382 34295171

[20] G. G. Munavalli , R. Guthridge , S. Knutsen‐Larson , A. Brodsky , E. Matthew , and M. Landau , “COVID‐19/SARS‐CoV‐2 Virus Spike Protein‐Related Delayed Inflammatory Reaction to Hyaluronic Acid Dermal Fillers: A Challenging Clinical Conundrum in Diagnosis and Treatment,” Archives of Dermatological Research 314 (2022): 1–15. 33559733 10.1007/s00403-021-02190-6 PMC7871141

[21] K. Beleznay , J. D. A. Carruthers , A. Carruthers , M. E. Mummert , and S. Humphrey , “Delayed‐Onset Nodules Secondary to a Smooth Cohesive 20 Mg/mL Hyaluronic Acid Filler: Cause and Management,” Dermatologic Surgery 41 (2015): 929–939. 26166260 10.1097/DSS.0000000000000418

[22] S. Humphrey , D. H. Jones , J. D. Carruthers , et al., “Retrospective Review of Delayed Adverse Events Secondary to Treatment With a Smooth, Cohesive 20‐Mg/mL Hyaluronic Acid Filler in 4500 Patients,” Journal of the American Academy of Dermatology 83 (2020): 86–95. 32035107 10.1016/j.jaad.2020.01.066

[23] T. Decates , J. Kadouch , P. Velthuis , and T. Rustemeyer , “Immediate Nor Delayed Type Hypersensitivity Plays a Role in Late Inflammatory Reactions After Hyaluronic Acid Filler Injections,” Clinical, Cosmetic and Investigational Dermatology 14 (2021): 581–589. 34103958 10.2147/CCID.S312198 PMC8178514

[24] A. A. Justiz Vaillant , R. Vashisht , and P. M. Zito , Immediate Hypersensitivity Reactions (2022), https://www.ncbi.nlm.nih.gov/books/NBK513315/. 30020687

[25] W. J. Pichler , “Delayed Drug Hypersensitivity Reactions,” Annals of Internal Medicine 139 (2003): 683–693. 14568857 10.7326/0003-4819-139-8-200310210-00012

[26] K. Sadtler , K. Estrellas , B. W. Allen , et al., “Developing a Pro‐Regenerative Biomaterial Scaffold Microenvironment Requires T Helper 2 Cells,” Science 1979, no. 352 (2016): 366–370. 10.1126/science.aad9272 PMC4866509 27081073

[27] S. D. Sommerfeld , C. Cherry , R. M. Schwab , et al., “Interleukin‐36γ–Producing Macrophages Drive IL‐17–Mediated Fibrosis,” Science Immunology 4 (2019): eaax4783. 31604843 10.1126/sciimmunol.aax4783 PMC7549193

[28] L. Chung , D. R. Maestas, Jr. , A. Lebid , et al., “Interleukin 17 and Senescent Cells Regulate the Foreign Body Response to Synthetic Material Implants in Mice and Humans,” Science Translational Medicine 12 (2020): eaax3799. 32295900 10.1126/scitranslmed.aax3799 PMC7219543

[29] J. S. Maltzman , H. O. Reed , and M. L. Kahn , “HA‐Ving Lymphatics Improves Lung Transplantation,” Journal of Clinical Investigation 125 (2015): 3999–4001. 26524589 10.1172/JCI84549 PMC4639989

[30] S. Abdalla , G. Makhoul , M. Duong , R. C. J. Chiu , and R. Cecere , “Hyaluronic Acid‐Based Hydrogel Induces Neovascularization and Improves Cardiac Function in a Rat Model of Myocardial Infarction,” Interactive Cardiovascular and Thoracic Surgery 17 (2013): 767–772. 23851989 10.1093/icvts/ivt277 PMC3805189

[31] R. C. Gupta , R. Lall , A. Srivastava , and A. Sinha , “Hyaluronic Acid: Molecular Mechanisms and Therapeutic Trajectory,” Frontiers in Veterinary Science 6 (2019): 1–24. 31294035 10.3389/fvets.2019.00192 PMC6603175

[32] J. S. Frenkel , “The Role of Hyaluronan in Wound Healing,” International Wound Journal 11 (2014): 159–163. 22891615 10.1111/j.1742-481X.2012.01057.x PMC7950635

[33] M. Sun , S. Puri , K. N. Mutoji , et al., “Hyaluronan Derived From the Limbus Is a Key Regulator of Corneal Lymphangiogenesis,” Investigative Ophthalmology & Visual Science 60 (2019): 1050–1062. 30897620 10.1167/iovs.18-25920 PMC6432804

[34] M. Wu , Y. du , Y. Liu , et al., “Low Molecular Weight Hyaluronan Induces Lymphangiogenesis Through LYVE‐1‐Mediated Signaling Pathways,” PLoS One 9 (2014): e92857. 24667755 10.1371/journal.pone.0092857 PMC3965470

[35] L. A. Johnson and D. G. Jackson , “Hyaluronan and Its Receptors: Key Mediators of Immune Cell Entry and Trafficking in the Lymphatic System,” Cells 10 (2021): 2061. 34440831 10.3390/cells10082061 PMC8393520

[36] M. I. Tammi , A. J. Day , and E. A. Turley , “Hyaluronan and Homeostasis: A Balancing Act,” Journal of Biological Chemistry 277 (2002): 4581–4584. 11717316 10.1074/jbc.R100037200

[37] A. D. Agostino , et al., “In Vitro Analysis of the Effects on Wound Healing of High‐ and Low‐Molecular Weight Chains of Hyaluronan and Their Hybrid H‐HA / L‐HA Complexes,” BMC Cell Biology 16 (2015): 1–15, 10.1186/s12860-015-0064-6. 26163378 PMC4499215

[38] X. Xu , A. K. Jha , D. A. Harrington , M. C. Farach‐Carson , and X. Jia , “Hyaluronic Acid‐Based Hydrogels: From a Natural Polysaccharide to Complex Networks,” Soft Matter 8 (2012): 3280–3294. 22419946 10.1039/C2SM06463D PMC3299088

[39] X. Li , Y. Shou , and A. Tay , “Hydrogels for Engineering the Immune System,” Advanced NanoBiomed Research 1 (2021): 2000073.

[40] W. Bu , Y. Wu , A. M. Ghaemmaghami , H. Sun , and A. Mata , “Rational Design of Hydrogels for Immunomodulation,” Regenerative Biomaterials 9 (2022): rbac009. 35668923 10.1093/rb/rbac009 PMC9160883

[41] T. Luo , B. Tan , L. Zhu , Y. Wang , and J. Liao , “A Review on the Design of Hydrogels With Different Stiffness and Their Effects on Tissue Repair,” Frontiers in Bioengineering and Biotechnology 10 (2022): 1–18. 10.3389/fbioe.2022.817391 PMC8822157 35145958

[42] A. Saradna , D. C. Do , S. Kumar , Q. L. Fu , and P. Gao , “Macrophage Polarization and Allergic Asthma,” Translational Research 191 (2018): 1–14. 29066321 10.1016/j.trsl.2017.09.002 PMC5776696

[43] K. Suzuki , K. Meguro , D. Nakagomi , and H. Nakajima , “Roles of Alternatively Activated M2 Macrophages in Allergic Contact Dermatitis,” Allergology International 66 (2017): 392–397. 28320580 10.1016/j.alit.2017.02.015

[44] K.‐A. Moon , S. Y. Kim , T. B. Kim , et al., “Allergen‐Induced CD11b+ CD11c(Int) CCR3+ Macrophages in the Lung Promote Eosinophilic Airway Inflammation in a Mouse Asthma Model,” International Immunology 19 (2007): 1371–1381. 17977814 10.1093/intimm/dxm108

[45] M. Utsugi , “Antigen Presenting Cells in Allergy,” Nippon Rinsho. Japanese Journal of Clinical Medicine 67 (2009): 2076–2081. 19899519

[46] S. J. Galli and M. Tsai , “IgE and Mast Cells in Allergic Disease,” Nature Medicine 18 (2012): 693–704. 10.1038/nm.2755 PMC3597223 22561833

[47] Y. Huang , N. Jin , C. L. Roark , et al., “The Influence of IgE‐Enhancing and IgE‐Suppressive γδ T Cells Changes With Exposure to Inhaled Ovalbumin,” Journal of Immunology 183 (2009): 849–855. 10.4049/jimmunol.0804104 PMC2712735 19542369

[48] Y. Huang , Z. Yang , J. McGowan , H. Huang , R. L. O'Brien , and W. K. Born , “Regulation of IgE Responses by γδ T Cells,” Current Allergy and Asthma Reports 15 (2015): 13. 26130476 10.1007/s11882-015-0519-z

[49] X. Huang , Q. Yang , Y. Chen , and G. Zhang , “Correlation of Gammadelta‐T‐Cells, Th17 Cells and IL‐17 in Peripheral Blood of Patients With Allergic Rhinitis,” Asian Pacific Journal of Allergy and Immunology 32 (2014): 235–239. 25268341 10.12932/AP0432.32.3.2014

[50] Z. W. Gu , Y. X. Wang , and Z. W. Cao , “Neutralization of Interleukin‐17 Suppresses Allergic Rhinitis Symptoms by Downregulating Th2 and Th17 Responses and Upregulating the Treg Response,” Oncotarget 8 (2017): 22361–22369. 28423590 10.18632/oncotarget.15652 PMC5410229

[51] A. M. Russano , E. Agea , L. Corazzi , et al., “Recognition of Pollen‐Derived Phosphatidyl‐Ethanolamine by Human CD1d‐Restricted γδ T Cells,” Journal of Allergy and Clinical Immunology 117 (2006): 1178–1184. 16675349 10.1016/j.jaci.2006.01.001

[52] P. W. Askenase , “Yes T Cells, but Three Different T Cells (αβ, γσ and NK T Cells), and Also B‐1 Cells Mediate Contact Sensitivity,” Clinical and Experimental Immunology 125 (2001): 345–350. 11531940 10.1046/j.1365-2249.2001.01619.x PMC1906150

[53] R. A. Campos , M. Szczepanik , M. Lisbonne , A. Itakura , M. Leite‐de‐Moraes , and P. W. Askenase , “Invariant NKT Cells Rapidly Activated via Immunization With Diverse Contact Antigens Collaborate In Vitro With B‐1 Cells to Initiate Contact Sensitivity,” Journal of Immunology 177 (2006): 3686–3694. 10.4049/jimmunol.177.6.3686 16951328

[54] H. Huber , P. Descossy , R. van Brandwijk , and J. Knop , “Activation of Murine Epidermal TCR‐Gamma Delta+ T Cells by Keratinocytes Treated With Contact Sensitizers,” Journal of Immunology 155 (1995): 2888–2894. 7673705

[55] M. Szczepanik , “Regulation of the Contact Sensitivity Reaction by Suppression of T Gamma Delta Lymphocytes,” Folia Medica Cracoviensia 39 (1998): 5–33. 10481375

[56] V. Mraz , C. Geisler , and C. M. Bonefeld , “Dendritic Epidermal T Cells in Allergic Contact Dermatitis,” Frontiers in Immunology 11 (2020): 1–9. 32508820 10.3389/fimmu.2020.00874 PMC7248261

[57] Y. Dong , A. Arif , M. Olsson , et al., “Endotoxin Free Hyaluronan and Hyaluronan Fragments Do Not Stimulate TNF‐α, Interleukin‐12 or Upregulate Co‐Stimulatory Molecules in Dendritic Cells or Macrophages,” Scientific Reports 6 (2016): 1–15, 36928. 27869206 10.1038/srep36928 PMC5116629

[58] C. Tang , M. D. Inman , N. van Rooijen , et al., “Th Type 1‐Stimulating Activity of Lung Macrophages Inhibits Th2‐Mediated Allergic Airway Inflammation by an IFN‐Gamma‐Dependent Mechanism,” Journal of Immunology 166 (2001): 1471–1481. 10.4049/jimmunol.166.3.1471 11160186

[59] L. K. Teixeira , B. P. F. Fonseca , B. A. Barboza , and J. P. B. Viola , “The Role of Interferon‐Gamma on Immune and Allergic Responses,” Memórias do Instituto Oswaldo Cruz 100, no. Suppl (2005): 137–144. 10.1590/s0074-02762005000900024 15962113

[60] H. Shen , P. Yao , E. Lee , D. Greenhalgh , and A. M. Soulika , “Interferon‐Gamma Inhibits Healing Post Scald Burn Injury,” Wound Repair and Regeneration 20 (2012): 580–591. 22712462 10.1111/j.1524-475X.2012.00812.x

[61] Y. Ishida , T. Kondo , T. Takayasu , Y. Iwakura , and N. Mukaida , “The Essential Involvement of Cross‐Talk Between IFN‐γ and TGF‐β in the Skin Wound‐Healing Process,” Journal of Immunology 172 (2004): 1848–1855. 10.4049/jimmunol.172.3.1848 14734769

[62] N. X. Landén , D. Li , and M. Ståhle , “Transition From Inflammation to Proliferation: A Critical Step During Wound Healing,” Cellular and Molecular Life Sciences 73 (2016): 3861–3885. 27180275 10.1007/s00018-016-2268-0 PMC5021733

[63] U. Wollina and A. Goldman , “Spontaneous and Induced Degradation of Dermal Fillers: A Review,” Journal of Cutaneous and Aesthetic Surgery 17 (2024): 273. 39649762 10.4103/JCAS.JCAS_137_23 PMC11619174

[64] J. M. Anderson , A. Rodriguez , and D. T. Chang , “Foreign Body Reaction to Biomaterials,” Seminars in Immunology 20 (2008): 86–100. 18162407 10.1016/j.smim.2007.11.004 PMC2327202

