# Supplementary material for: Innate and Adaptive Immune Responses to Clinical Hyaluronic Acid Fillers
Source: J Cosmet Dermatol. 2025 Jul 10;24(7):e70292. doi: 10.1111/jocd.70292 (PMC12242719; doi:10.1111/jocd.70292)
Supplement: Supplementary file 1 — Figure S1. Additional muscle immune cell populations at 6 weeks following VML. PMN, polymorphonuclear leukocyte. (Statistics) Data are mean ± SD, n = 4, ****p < 0.0001, ***p < 0.001, **p < 0.01, and *p < 0.05 by one‐way ANOVA with Tukey’s multiple comparisons test. Figure S2. Gating scheme for Aurora Pan‐Immune panel on quadricep muscle. Figure S3. Gating scheme for Attune myeloid panel on quadricep muscle. Figure S4. Gating scheme for Attune ICS panel on quadricep muscle. Figure S5. Gating scheme for Attune ICS panel on inguinal lymph node. [file JOCD-24-e70292-s001.docx]

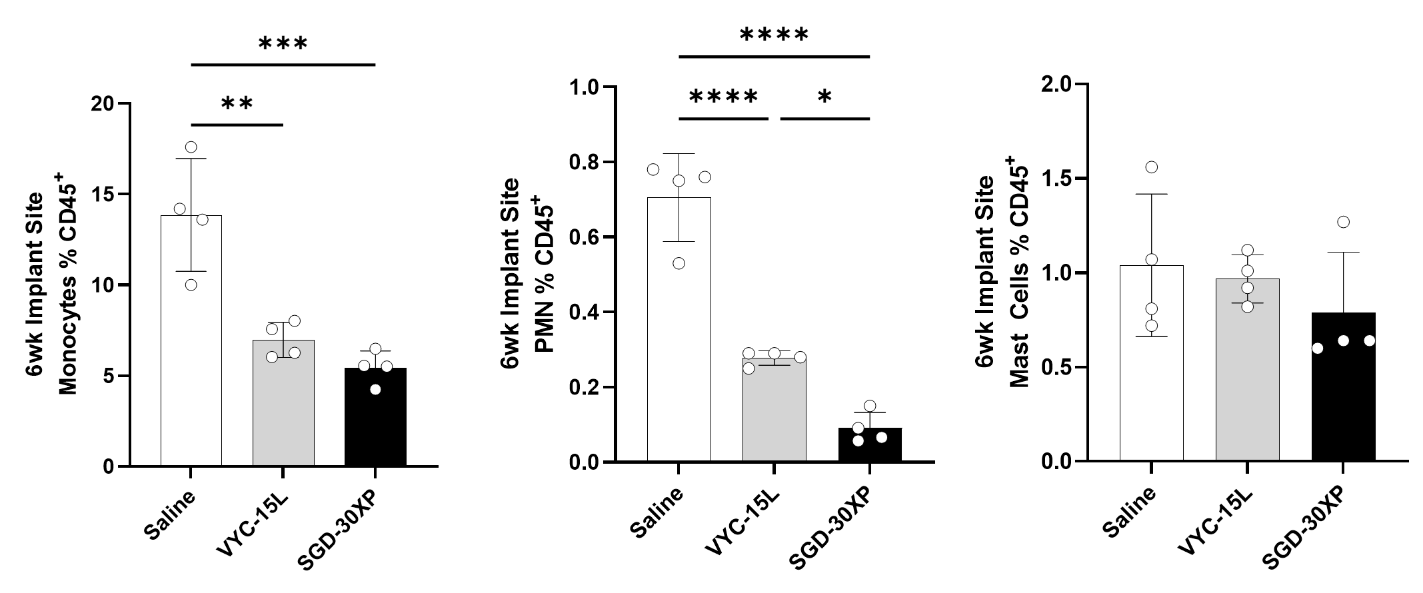


**Supplemental Figure 1**. Additional muscle immune cell populations at 6 weeks following VML. PMN, polymorphonuclear leukocyte. **(Statistics)** Data are mean ± SD, n = 4, ****P < 0.0001, ***P < 0.001, **P < 0.01, and *P < 0.05 by one-way ANOVA with Tukey's multiple comparisons test.


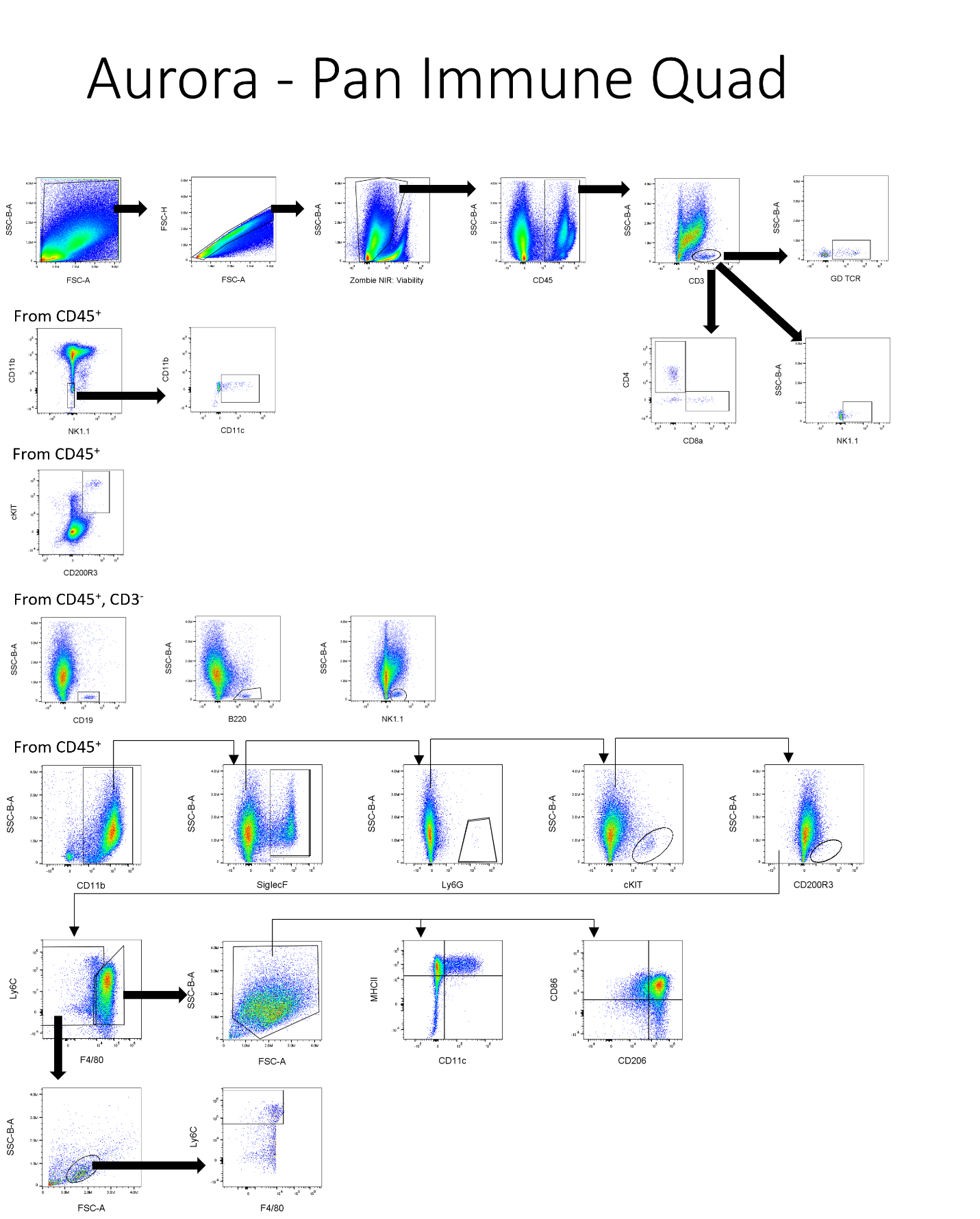


**Supplemental Figure 2**. Gating scheme for Aurora Pan-Immune panel on quadricep muscle.


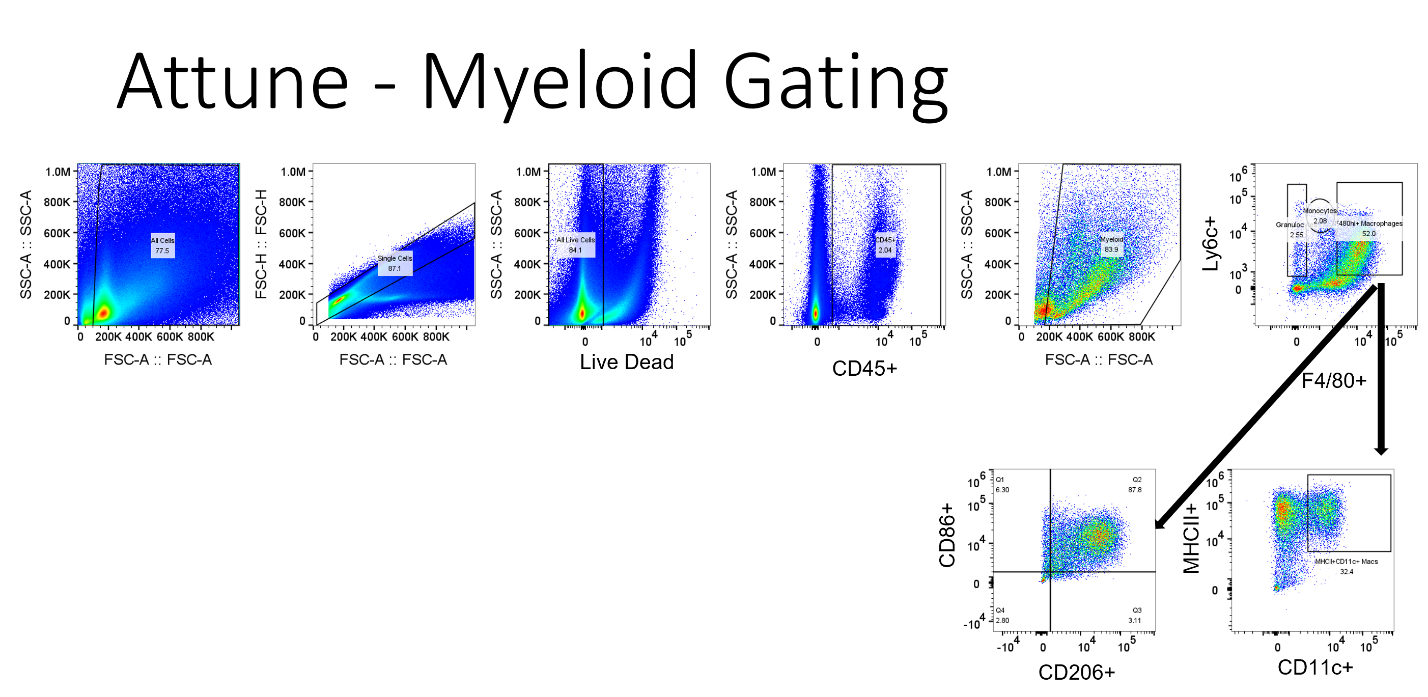


**Supplemental Figure 3**. Gating scheme for Attune myeloid panel on quadricep muscle.


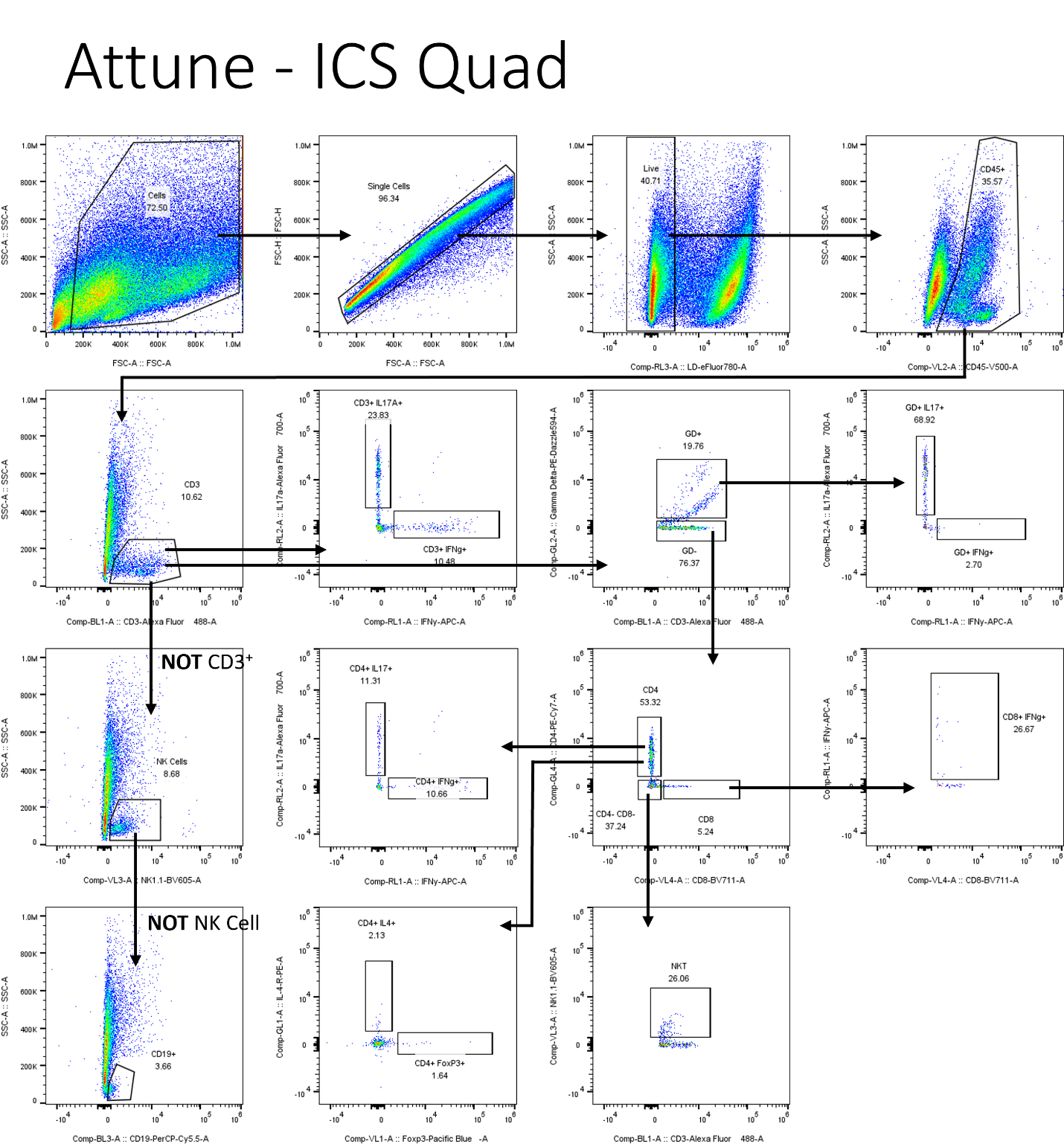


**Supplemental Figure 4**. Gating scheme for Attune ICS panel on quadricep muscle.


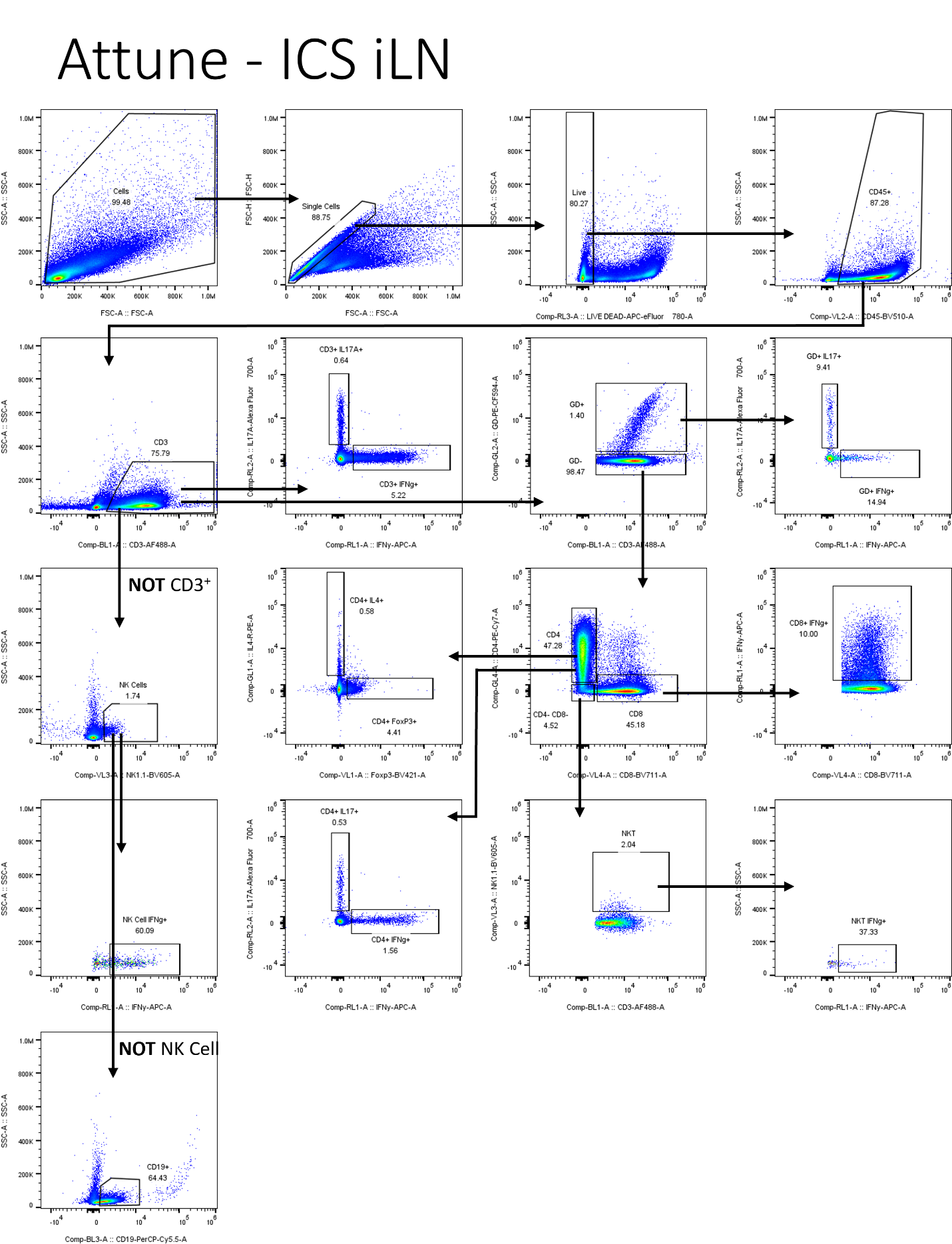


**Supplemental Figure 5**. Gating scheme for Attune ICS panel on inguinal lymph node.
